# Venous beading in two or more quadrants might not be a sensitive grading criterion for severe nonproliferative diabetic retinopathy

**DOI:** 10.1007/s00417-018-3971-3

**Published:** 2018-04-06

**Authors:** Ling Chen, Xiongze Zhang, Feng Wen

**Affiliations:** 1 State Key Laboratory of Ophthalmology, Zhongshan Ophthalmic Center, Sun Yat-sen University, Guangzhou, 510060 China; 2 Present Address: Guangzhou, China

**Keywords:** Diabetic retinopathy, Severe nonproliferative diabetic retinopathy, Proliferative diabetic retinopathy, Retinal vascular lesions, Venous beading, Grading

## Abstract

**Purpose:**

To determine whether venous beading (VB) in two or more quadrants is an appropriate grading criterion for severe nonproliferative diabetic retinopathy (NPDR).

**Methods:**

A hospital-based, retrospective, cross-sectional study. A total of 806 patients admitted with diabetic retinopathy (DR) from January 2014 to April 2017 were included in this study. DR severity was graded by the international grading criterion. The status of VB, intraretinal microvascular abnormalities (IRMA), capillary nonperfusion, arteriovenous nicking, and diabetic macular edema was evaluated based on fundus fluorescein angiography.

**Results:**

The prevalence of VB in eyes with proliferative diabetic retinopathy (PDR), severe NPDR, and moderate NPDR was 41.3% (327/791), 5.9% (31/526), and 0% (0/295), respectively (*p* < 0.001). Moreover, the proportion of VB in two or more quadrants was even lower (27.1% for PDR and 2.1% for severe NPDR, *p* < 0.001), and among the total of 225 eyes with VB in two or more quadrants, 214 eyes (95.1%) were graded as PDR. Furthermore, VB formation was significantly correlated with capillary nonperfusion, duration of diabetes (both *p* < 0.001), and smoking (*p* < 0.05). After adjusting for age, sex, and other possible factors, VB (OR = 7.479, *p* < 0.001) and IRMA (OR = 2.433, *p* < 0.001) were determined as independent risk factors for developing PDR.

**Conclusions:**

Our study suggested that VB in two or more quadrants might not be a sensitive grading criterion for severe NPDR among a Chinese population with type 2 diabetes. Nevertheless, VB has a great specificity to define an advanced form of DR.

## Introduction

Diabetic retinopathy (DR) is one of the most common microvascular complications of diabetes mellitus (DM); worldwide, DR has become the leading cause of blindness in working-age adults, especially in Asian countries due to the sharply increasing prevalence of type 2 diabetes [1–3]. DR is a preventable blinding eye disease but is unfortunately usually overlooked at the early stages. Once the disease progresses to a severe stage, such as proliferative DR (PDR), visual impairment may be inevitable [4]. Although laser photocoagulation and anti-VEGF injections are recognized as effective treatments for PDR, these treatments rarely restore vision [5, 6]. Therefore, early screening for DR and appropriate disease severity grading as well as timely intervention are vital to reduce the incidence of sight-threatening visual impairment [7–9].

DR severity grading was conventionally based on standard, seven-field stereoscopic photographs according to the Early Treatment of Diabetic Retinopathy Study (ETDRS) [8]. Subsequently, the International Diabetic Retinopathy Severity Scales (IDRSS) were proposed as a simplified grading version [10] and approved as the international DR grading criterion by the Diabetic Retinopathy Preferred Practice Pattern (DR PPP) [11]. This criterion divided DR into the following levels with increasing risks of retinopathy: (1) no apparent retinopathy, (2) mild nonproliferative DR (NPDR), (3) moderate NPDR, (4) severe NPDR, and (5) PDR. The determination of severe NPDR, which is currently based on the international 4-2-1 rule (intraretinal hemorrhage (IRH), venous beading (VB), and intraretinal microvascular abnormalities (IRMA)), is quite critical due to its high probability of progressing to PDR [9]. However, we found that VB was less commonly observed in severe NPDR eyes in our clinical practice, and most eyes with VB in two or more quadrants had progressed to PDR. Moreover, the prevalence of venous abnormalities including VB in patients with type 2 diabetes reportedly varies among ethnic groups [12]. And the prevalence of retinal vascular lesions in different DR severity levels has not been reported.

In the present study, we determined the prevalence of retinal vascular lesions among different DR severity levels and assessed whether VB in two or more quadrants was an appropriate grading criterion for severe NPDR among a Chinese population with type 2 diabetes, using a combination of dilated ophthalmoscopy and fundus fluorescein angiography (FFA).

## Materials and methods

### Patients

The present study was a hospital-based, retrospective, cross-sectional study. A total of 1264 patients with type 2 diabetes were diagnosed with DR and referred to the Zhongshan Ophthalmic Center for FFA examination from January 2014 to April 2017. Patients who had a previous ophthalmological intervention procedure, such as laser photocoagulation, vitrectomy, or anti-VEGF injection in one or both eyes, were excluded from this study. In addition, eyes with other concomitant retinal diseases or poor-quality images were excluded. After these criteria were applied, 806 DR patients (1612 eyes) were eligible for inclusion in this study. The study protocol was conducted in accordance with the Declaration of Helsinki for research involving human subjects and was approved by the Institutional Review Board of the Zhongshan Ophthalmic Center at Sun Yat-sen University. Potential risks associated with the FFA examination were fully discussed with the patients, and written informed consent was obtained from all included patients.

### Data collection

Demographic and lifestyle information, including age, sex, current smoking, current drinking, hypertension, age of DM onset and duration of diabetes, and ophthalmological medical histories, were obtained from a medical record review. All patients underwent a comprehensive ophthalmological examination, including measurement of visual acuity, slit lamp biomicroscopy, fundus examination, and FFA through dilated pupils. The DR disease severity level was graded according to findings observable on dilated ophthalmoscopy by two ophthalmologists (Ling Chen and Xiongze Zhang) based on the International Clinical Diabetic Retinopathy Disease Severity Scales [10]. If grading differed between the two ophthalmologists, consensus grading was conducted during a meeting with both ophthalmologists and a retinal specialist (Feng Wen). In addition, the DR severity level was corrected according to the FFA, which detected the vascular lesions more accurately [13].

### FFA

FFA images were obtained with a Heidelberg Retina Angiograph (HRA, Heidelberg Engineering, Heidelberg, Germany). Ten fields of 55° FFA images were taken from both eyes of each patient at the early and midterm stage after fluorescein injection. The ten fields were centered on the following areas: the macula, optic disc, superior peripheral retina, superior temporal peripheral retina, temporal peripheral retina, inferior temporal peripheral retina, inferior peripheral retina, inferior nasal peripheral retina, nasal, and superior nasal peripheral retina. At the late stage of FFA (approximately 15 min after fluorescein injection), 55° FFA images centered on the macula were taken of both the left and right eyes. The status of VB, IRMA, capillary nonperfusion, arteriovenous nicking, and diabetic macular edema (DME) were evaluated based on FFA images by two ophthalmologists who were blind to the DR stages. When patients underwent two or more FFA examinations, only the first one was recorded.

### Statistics

Statistical analyses were carried out with SPSS (SPSS 21.0). Descriptive statistics (means ± standard deviations [SDs]) of normally distributed variables and geometric means with 95% confidence intervals (CIs) of non-normally distributed variables were calculated. Differences in the prevalence of retinal vascular lesions among different DR severity levels were estimated by multiple logistic regression analyses, with adjustments for age, sex, and hypertension. Differences in the characteristics between patients with and without VB were assessed by multiple logistic regression analyses, with adjustments for age and sex. Multiple logistic regression models adjusted for age and sex were constructed to assess the risk factors of moderate NPDR progression to severe retinopathy. Odds ratios (ORs) and 95% CIs are presented. Significant differences were defined as *p* < 0.05.

## Results

A total of 1264 DR patients were referred to the Zhongshan Ophthalmic Center for FFA examination between January 2014 and April 2017. Among them, 211 individuals were excluded due to previous laser photocoagulation, 34 individuals were excluded for previous vitrectomy, 17 individuals were excluded due to previous anti-VEGF injection, 26 individuals were excluded for poor-quality images, and 170 individuals were excluded due to other concomitant retinal diseases. In the final analysis, 806 DR patients (1612 eyes) were eligible for inclusion in this study for evaluation.

### Clinical characteristics of patients included in the study

As shown in Table 1, a total of 791 eyes (49.1% [95% CI 46.8–53]) were graded as PDR, 526 eyes (32.6% [30.5–36]) were graded as severe NPDR, and 295 eyes were graded as moderate NPDR (18.3% [13.3–20.1]) according to the combination of findings observable on dilated ophthalmoscopy and FFA. The average age was 55.6 ± 7.7 for PDR, 59.8 ± 9.7 for severe NPDR, and 59.3 ± 9.5 for moderate NPDR patients. The age of DM onset was 46.1 ± 6.3 for PDR, 55.2 ± 9.7 for severe NPDR, and 55.6 ± 9.6 for moderate NPDR. Therefore, the duration of diabetes obtained by calculation was 9.3 ± 3.2, 4.6 ± 2.1, and 4.0 ± 2.4 for PDR, severe NPDR, and moderate NPDR, respectively. Furthermore, there were more males than females in the severe retinopathy stages (PDR and severe NPDR), while there was an approximately equal proportion of men and women in moderate NPDR. The proportion of hypertension in DR patients was 42.7% for PDR, 46.6% for severe NPDR, and 38.6% for moderate NPDR. In addition, logMAR vision was 0.73 ± 0.44, 0.58 ± 0.41, and 0.35 ± 0.29 for PDR, severe NPDR, and moderate NPDR, respectively. The proportion of current smoking in DR, severe NPDR, and moderate NPDR was 23.8, 22.4, and 17.6%, respectively, and the proportion of current drinking in the DR, severe NPDR, and moderate NPDR was 14.9, 13.3, and 11.5%, respectively.

**Table 1:** Demographic and clinical characteristics of patients included in the study

	PDR	Severe NPDR	Moderate NPDR
*n* (eyes)	791	526	295
Age (years)	55.6 ± 7.7	59.8 ± 9.7	59.3 ± 9.5
Age of DM onset (years)	46.1 ± 6.3	55.2 ± 9.7	55.6 ± 9.6
Duration of diabetes (years)	9.3 ± 3.2	4.6 ± 2.1	4.0 ± 2.4
Female sex (%)	46.9	40.9	50.2
Hypertension (%)	42.7	46.6	38.6
logMAR vision	0.73 ± 0.44	0.58 ± 0.41	0.35 ± 0.29
Current smoking (%)	23.8	22.4	17.6
Current drinking (%)	14.9	13.3	11.5

Data are *n*, means ± SD, or percentage

*PDR* proliferative diabetic retinopathy, *NPDR* nonproliferative diabetic retinopathy, *DM* diabetes mellitus

### Retinal vascular lesions

Table 2 shows the prevalence of retinal vascular lesions, including VB, IRMA, capillary nonperfusion, arteriovenous nicking, and DME, among the different DR severity levels based on FFA. Possible confounders, such as age, sex, and hypertension, were adjusted. VB was observed in 41.3% of the PDR eyes and 5.9% of the severe NPDR eyes, while no VB was detected in moderate NPDR eyes (*p* < 0.001, multiple logistic regression analyses). Additionally, the percentage of eyes in which VB was observed in ≥ 2 quadrants was 27.1% for PDR and 2.1% for severe NPDR (*p* < 0.001). Furthermore, among the total of 225 eyes with VB in two or more quadrants, 214 eyes (95.1%) were graded as PDR. In addition, the prevalence of IRMA was 81.5% in PDR and 53% in severe NPDR (*p* < 0.001). The prevalence of capillary nonperfusion was 97.1% for PDR, 84.6% for severe NPDR, and 28.1% for moderate NPDR (*p* < 0.001). Additionally, the prevalence of arteriovenous nicking was 28.6, 23, and 16.6% for PDR, severe NPDR, and moderate NPDR, respectively. The prevalence of DME was 30, 25.3, and 10.5% in PDR, severe NPDR, and moderate NPDR, respectively (*p* = 0.004, and *p* < 0.001, respectively).

**Table 2:** Prevalence of retinal vascular lesions by diabetic retinopathy severity levels

	PDR	Severe NPDR	Moderate NPDR	*p* value*
*n* (eyes)	791	526	295	
VB (%)	41.3	5.9	0	< 0.001
VB ≥ 2 quadrants (%)	27.1	2.1	0	< 0.001
IRMA (%)	81.5	53.0	0	< 0.001
Capillary nonperfusion (%)	97.1	84.6	28.1	< 0.001
Arteriovenous nicking (%)	28.6	23.0	16.6	0.004
DME (%)	30.0	25.3	10.5	< 0.001

Data are *n* or percentage

*PDR* proliferative diabetic retinopathy, *NPDR* nonproliferative diabetic retinopathy, *VB* venous beading, *IRMA* intraretinal microvascular abnormalities, *DME* diabetic macular edema

*Adjusted for age, sex, and hypertension

### Possible risk factors for VB formation

To determine the possible risk factors for VB formation, eyes were divided into the following two groups: group 1 with VB (*n* = 356) and group 2 without VB (*n* = 1256). Capillary nonperfusion, the age of DM onset, the duration of diabetes, and other possible factors were evaluated. Findings (Table 3) showed that the average age of DM onset in group 1 was 45.6 ± 7.0, which was significantly younger than that in group 2 (52.3 ± 9.5) (*p* < 0.001). The duration of diabetes in group 1 was 8.4 ± 3.0 and significantly longer than that group 2 (6.3 ± 3.7) (*p* < 0.001). Moreover, capillary nonperfusion was significantly correlated with VB formation (*p* < 0.001), and the proportion of smoking in patients in group 1 was significantly higher than that in patients in group 2 (*p* < 0.05). Additionally, the findings revealed no significant differences in the prevalence of current drinking, hypertension, and arteriovenous nicking between eyes with and without VB after adjustment for age and sex.

**Table 3:** Possible risk factors for venous beading formation

	With venous beading	Without venous beading	*p* value*
*n* (eyes)	356	1256	
Age of DM onset (years)	45.6 ± 7.1	52.3 ± 9.4	< 0.001
Duration of diabetes (years)	8.4 ± 2.9	6.3 ± 3.7	< 0.001
Current smoking (%)	28.5	20.2	0.017
Current drinking (%)	17.7	12.7	0.532
Hypertension (%)	39.3	44.3	0.402
Arteriovenous nicking (%)	28.7	23.4	0.068
Capillary nonperfusion (%)	98.9	75.2	< 0.001

Data are *n*, means ± SD, or percentage

*DM* diabetes mellitus

*Adjusted for age and sex

### Predictors for NPDR progression to PDR

The associations among possible risk factors, such as VB, IRMA, the age of DM onset, duration of DM, hypertension, arteriovenous nicking, DME, logMAR vision, capillary nonperfusion, current smoking, and current drinking, with the progression of NPDR to PDR, were assessed (Table 4). Multiple logistic regression models adjusted for age and sex were constructed. Results showed that the presence of VB increased the progression risk of NPDR to PDR by approximately 7.479 times (adjusted odds ratio 7.479; 95% CI 4.352–12.853) (*p* < 0.001). The presence of IRMA increased the progression risk of NPDR by approximately 2.433 times (adjusted ORs 2.433; 95% CI 1.630–3.630) (*p* < 0.001). And the presence of arteriovenous nicking also increased the progression risk of NPDR by approximately 2.889 times (adjusted ORs 2.889; 95% CI 1.813–4.602) (*p* < 0.001). Moreover, the ORs for NPDR progression to PDR were 0.870 per year of DM onset age (95% CI 0.838–0.902), 2.064 per year of diabetes duration (1.880–2.266), and 1.140 per 0.1 logMAR vision (1.086–1.197) (all *p* < 0.001). Additionally, patients with hypertension were more likely to progress to PDR than those without hypertension (adjusted odds ratio 1.967; 95% CI1.309–2.956) (*p* = 0.001).

**Table 4:** Predictors for NPDR progression to PDR

	*β*	OR [95% CI]	*p* value*
Venous beading	2.012	7.479 [4.352–2.853]	< 0.001
IRMA	0.889	2.433 [1.630–3.630]	< 0.001
Age of DM onset	− 0.140	0.870 [0.838–0.902]	< 0.001
Duration of diabetes	0.725	2.064 [1.880–2.266]	< 0.001
Hypertension	0.677	1.967 [1.309–2.956]	0.001
Arteriovenous nicking	1.061	2.889 [1.813–4.602]	< 0.001
logMAR vision (per 0.1)	0.131	1.140 [1.086–1.197]	< 0.001

Data are *β* and ORs

*IRMA* intraretinal microvascular abnormalities, *DM* diabetes mellitus *Adjusted for age and sex

## Discussion

The present study determined the prevalence of retinal vascular lesions, including VB, IRMA, capillary nonperfusion, arteriovenous nicking, and DME, among different DR severity levels based on a combination of dilated ophthalmoscopy and FFA. In addition, the independent, relevant factors for VB formation and NPDR progression to PDR were assessed.

In this study, retinal vascular lesions were evaluated based on FFA, allowing the detection of subtle lesions [14]. Clinically, small retinal vascular lesions are easily overlooked by direct ophthalmoscopy [15], but FFA significantly improves the detection rate of retinal vascular lesions [16]. The prevalence of VB in our study was lower than that reported in Japanese DR patients [17]. A previous study showed that more VB was observed in Chinese DM patients than in Caucasian patients (8.7 vs. 2.1%) [12];nevertheless, there were no data regarding its prevalence among different DR severity levels. According to DR PPP, VB in two or more quadrants on dilated ophthalmoscopy is the international grading criterion for severe NPDR. However, only 2.1% of the severe NPDR eyes presented with VB in two or more quadrants in this study. We speculated that the prevalence of VB assessed by direct ophthalmoscopy would be even lower. Furthermore, among the total of 225 eyes with VB in two or more quadrants, 214 eyes (more than 95%) had entered the PDR stage. Therefore, we suggested that VB in two or more quadrants might be a too strict criterion for judging severe NPDR, at least among a Chinese population with type 2 DM. No VB was observed even with FFA in moderate NPDR eyes, indicating that once VB is observed, DR has progressed to the advanced stage (severe NPDR or PDR). Our results indicated that although VB in two or more quadrants might not be considered a sensitive criterion for judging severe NPDR, VB had a great specificity to define an advanced form of DR.

To the best of our knowledge, there were no data regarding the prevalence of IRMA among different DR severity levels. Our results showed that 53% of the severe NPDR had IRMA, which was far higher than the proportion of VB in severe NPDR. Previous clinicopathologic study demonstrated that IRMA had the particular potential for neovascularization [18]. Recent study suggested that IRMA significantly increased risk of PDR [19]. Additionally, our study also contributed data regarding the prevalence of other retinal vascular lesions, including capillary nonperfusion, arteriovenous nicking, and DME, among different DR severity levels.

In addition, our study revealed that the formation of VB was significantly correlated with age of DM onset, diabetes duration, capillary nonperfusion, and smoking, while it was not associated with drinking, hypertension, and arteriovenous nicking. VB is the chronic reactive expansion of the retinal vein in response to retinal ischemia or other abnormal situations [20], although idiopathic VB can occur in some cases [21]. As DR progresses, the death of pericytes and basement membrane thickening result in impaired perfusion and retinal ischemia, while increased ischemia leads to the formation of VB [22]. Indeed, our study suggested that the formation of VB was significantly associated with capillary nonperfusion and the disease course of DR, especially the age of DM onset and diabetes duration.

Additionally, VB, IRMA, duration of diabetes, hypertension, logMAR vision, and arteriovenous nicking were determined as independent risk factors for NPDR progression to PDR after adjustment for age and sex, while the age of DM onset was determined as an independent protective factor, which was consistent with previous studies [19, 23–26]. Furthermore, our results indicated that VB and IRMA were important predictors of NPDR progression to PDR, and the ORs of VB were even greater than that of IRMA. Moreover, we should pay more attention to visual changes, blood pressure, and arteriovenous nicking of DR patients. And patients with a long duration of diabetes and younger age of DM onset must undergo regular fundus exams. Nevertheless, in our study, a considerable proportion of patients had progressed to PDR (49.1%) when they were referred to the ophthalmologist. Early detection for retinopathy contributes to the reduction of the occurrence of severe DR [27, 28], but unfortunately, a significant proportion of type 2 DM patients does not undergo early retinal screening in China, especially in rural areas, before the onset of severe visual impairment [29], which could explain the high proportion of severe NPDR and PDR in our study.

There are several limitations to our study that need to be considered. Firstly, as a clinic-based retrospective study, selection bias may have occurred when patients were recruited into this study. Secondly, all patients were from a single institution, so a referral bias may exist. Thirdly, we did not evaluate the prevalence of “IRH in each of four quadrants,” because FFA was mainly evaluated in our study and it showed no advantage regarding the detection of IRH. Nevertheless, our study for the first time reported the prevalence of retinal vascular lesions among different DR severity levels based on FFA. And our study also contributed to explore more appropriate DR disease severity grading criteria and disease progression relevant factors.

In conclusion, our study suggested that VB in two or more quadrants might not be a sensitive grading criterion for severe NPDR in a Chinese population with type 2 diabetes. A more sensitive criterion for severe NPDR should be explored in the future to reduce the incidence of sight-threatening visual impairment. Nevertheless, VB has a great specificity to define an advanced form of DR.
